# Cardiac and hepatic iron and ejection fraction in thalassemia major: Multicentre prospective comparison of combined Deferiprone and Deferoxamine therapy against Deferiprone or Deferoxamine Monotherapy

**DOI:** 10.1186/1532-429X-15-1

**Published:** 2013-01-16

**Authors:** Alessia Pepe, Antonella Meloni, Giuseppe Rossi, Liana Cuccia, Giuseppe Domenico D’Ascola, Michele Santodirocco, Paolo Cianciulli, Vincenzo Caruso, Maria Antonietta Romeo, Aldo Filosa, Lorella Pitrolo, Maria Caterina Putti, Angelo Peluso, Saveria Campisi, Massimiliano Missere, Massimo Midiri, Letizia Gulino, Vincenzo Positano, Massimo Lombardi, Paolo Ricchi

**Affiliations:** 1 Cardiovascular MR Unit, Fondazione G, Monasterio CNR-Regione Toscana and Institute of Clinical Physiology, Pisa, Italy; 2 Epidemiology and Biostatistics Unit, Institute of Clinical Physiology, CNR, Pisa, Italy; 3 Ematologia-Emoglobinopatie, Civico Hospital - ARNAS, Palermo, Italy; 4 U.O. Microcitemie, A. O, ”Bianchi-Melacrino-Morelli”, Reggio Calabria, Italy; 5 Centro Microcitemia – D.H. Thalassemia Poliambulatorio “Giovanni Paolo II”, Ospedale Casa Sollievo della Sofferenza IRCCS, Opera di Padre Pio da Pietrelcina, San Giovanni Rotondo, Italy; 6 Centro Talassemie, “Sant′Eugenio” Hospital, Roma, Italy; 7 Centro Microcitemia, “Garibaldi” Hospital, Catania, Italy; 8 Department of Pediatrics, University of Catania, Catania, Italy; 9 Centro per la Cura delle Microcitemie, Cardarelli Hospital, Napoli, Italy; 10 U.O. Pediatria II, Az. Osp. “Villa Sofia”, Palermo, Italy; 11 Department of Pediatrics, University of Padova, Padova, Italy; 12 Microcitemia - Azienda Unità Sanitaria Locale TA/1, Presidio Ospedaliero Centrale, Taranto, Italy; 13 U.O.S. Talassemia, A.O. Umberto I, Siracusa, Italy; 14 Radiology Department, “John Paul II” Catholic University, Campobasso, Italy; 15 Department of Radiology, University of Palermo, Palermo, Italy

**Keywords:** Thalassemia, Chelation therapy, Cardiovascular magnetic resonance

## Abstract

**Background:**

Due to the limited data available in literature, the aim of this multi-centre study was to prospectively compare in thalassemia major (TM) patients the efficacy of combined deferiprone (DFP) and deferoxamine (DFO) regimen versus either DFP and DFO in monotherapy by cardiovascular magnetic resonance (CMR) over a follow up of 18 months.

**Methods:**

Among the first 1135 TM patients in the MIOT (Myocardial Iron Overload in Thalassemia) network, we evaluated those who had received either combined regimen (DFO + DFP, N=51) or DFP (N=39) and DFO (N=74) monotherapies between the two CMR scans. Iron overload was measured by T2* multiecho technique. Biventricular function parameters were quantitatively evaluated by cine images.

**Results:**

The percentage of patients that maintained a normal global heart T2* value was comparable between DFP+DFO versus both monotherapy groups. Among the patients with myocardial iron overload at baseline, the changes in the global heart T2* and in biventricular function were not significantly different in DFP+DFO compared with the DFP group. The improvement in the global heart T2* was significantly higher in the DFP+DFO than the DFO group, without a difference in biventricular function. Among the patients with hepatic iron at baseline, the decrease in liver iron concentration values was significantly higher with combination therapy than with either monotherapy group.

**Conclusions:**

In TM patients at the dosages used in the real world, the combined DFP+DFO regimen was more effective in removing cardiac iron than DFO, and was superior in clearing hepatic iron than either DFO or DFP monotherapy. Combined therapy did not show an additional effect on heart function over DFP.

## Background

To date one of the most imperative need in the field of thalassemia major (TM) is to treat patients with tailored chelation therapy. Thus, there is the need of comparing the efficacy of each chelator using standardized and validated techniques as a means of assessing changes in cardiac iron and function and in liver siderosis. In the current clinical practice removal of iron overload can be achieved using subcutaneous deferoxamine (DFO), oral deferiprone (DFP), oral deferasirox (DFX), as well as combination therapy with deferiprone and deferoxamine (DFP+DFO). A large body of evidence is present in literature about the comparison between DFP and DFO in monotherapy [1-3]. However, few studies have compared each chelator with their combinations in terms of differential effect in removing myocardial or liver iron and in improving cardiac function using T2* magnetic resonance imaging (MRI) or other validated procedures. In 2007 a randomized placebo controlled study from Sardinia demonstrated that combination therapy DFP+DFO was significantly more effective than DFO in clearing myocardial iron and in improving left ventricular (LV) ejection fraction (EF) in mild to moderate iron loaded patients [4]. In the same population a prospective study showed that combined chelation therapy DFP+DFO effectively reduced myocardial iron and improved cardiac function in patients with severe myocardial siderosis and impaired LV function [5]. Another small sized one-year study using changes in liver iron concentration (LIC) assessed by biopsies showed the superiority of the combined DFP+DFO therapy with respect to the DFP alone [6]. More recently, a retrospective observational study from Greece seemed to confirm that combination therapy was the most rapid chelation regimen able to improve hepatic and cardiac T2* values [7]. No long term data are available in literature about observational prospective comparisons of the effects on cardiac iron and function and liver iron in TM patients treated with combined DFP+DFO versus DFP in monotherapy.

Cardiovascular magnetic resonance (CMR) is ideally suited to compare different chelation treatments, providing highly reproducible and non-invasive measurements of myocardial [8-10] and liver iron burden [11,12] by the T2* technique. Moreover, CMR is the gold standard in cardiology for quantifying biventricular function parameters [13].

As randomized clinical trials are expensive to perform, networks of thalassemia and CMR centers, that have agreed to standardize and share their clinical and instrumental data, are powerful and recommended to evaluate chelation regimens in the real life.

The aim of this multi-centre study was to assess prospectively in a large clinical setting of TM patients the efficacy of combined DFP+DFO versus DFP and versus DFO in monotherapy by quantitative CMR over a follow up of 18 months.

## Methods

### Study population

The MIOT (Myocardial Iron Overload in Thalassemia) project is an Italian network built in 2006 and constituted at the time of this study by 68 thalassemia centres and 8 CMR centres where CMR exams are performed using homogeneous, standardized and validated procedures [14]. All centres are linked by a web-based network, configured to collect and share patients^′^ data [15].

**Figure 1:**
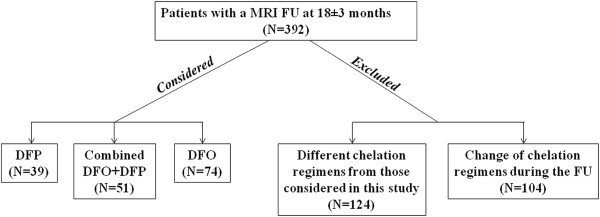
The patient flow.

Among the first 1135 TM patients enrolled in the MIOT project, 392 performed a CMR follow up study at 18 ± 3 months, according to the protocol. One hundred and four patients in any treatment regimen were excluded because they changed the chelation during the follow up time due to clinical reasons. Moreover, none of these patients repeated the CMR exam before the modification of the therapy due to logistic reasons. One hundred and twenty four patients were not considered because they received between the two CMR scans different chelation regimens from those considered in the present study. So, we evaluated prospectively the 164 TM patients who had been maintained combined DFP+DFO therapy or DFP and DFO in monotherapy between the two CMR scans (according to the protocol any change in the chelation regimen was made within one month after the CMR scan). Thus, we identified 3 groups of patients: 51 treated with combined DFP+DFO, 39 treated with DFP and 74 treated with DFO. Figure 1 shows the patient flow.

Among the 51 patients treated with DFP+DFO, 49 were in DFP+DFO before the first CMR scan and two switched to DFP+DFO after the first CMR (1 patient from DFP, 1 patient from DFX). Among the 39 patients treated with DFP, 35 were in DFP before the first CMR scan and 4 switched to DFP after the first CMR scan (2 patients from combined DFP+DFO, 1 patient from sequential DFP-DFO and 1 patient from deferasirox). Among the 74 patients treated with DFO, 71 were in DFO before the first CMR scan and 3 switched to DFO after the first CMR scan (1 patient from DFP, 1 patient from DFX and 1 patient from sequential DFP/DFX). All chelation regimens were prescribed based on the current clinical practice according to clinical/laboratory and instrumental data.

All patients had been regularly transfused since early childhood and started chelation therapy from the mid-to-late 1970s on, while patients born after the 1970s have received chelation therapy since early childhood. CMR was performed within 1 week before the regularly scheduled blood transfusion.

The study complied with the Declaration of Helsinki. All patients gave written informed consent to the protocol. The institutional review board approved this study.

### Magnetic resonance imaging

At all eight centres, CMR was performed using a 1.5 T scanner (GE Signa/Excite HD, Milwaukee, WI, USA). An eight-element cardiac phased-array receiver surface coil with breath-holding in end-expiration and ECG-gating was used for signal reception.

The T2* technique was used for iron overload assessment. Its reproducibility and its transferability within the MIOT network had been previously demonstrated for the heart and for the liver [14]. For the heart, a multislice multiecho T2* approach was used. Three parallel short-axis views (basal, medium and apical) of the LV were obtained. Each single short-axis view was acquired at nine echo times (TEs). Acquisition sequence details are provided in [3,9,16]. For the liver a single transverse slice was obtained at nine TEs using a T2* gradient–echo multiecho sequence in a standard way [11]. T2* images analysis was performed using a custom-written, previously validated software program (HIPPO MIOT®, IFC-CNR) [16]. The software plot the myocardial T2* distribution into a 16-segment LV model according to the American Heart Association (AHA)/American College of Cardiology (ACC) standardized myocardial segmentation [17]. For the heart an exponential + truncation curve fitting model was applied [16]. The global heart T2* value was obtained by averaging all segmental T2* values and the T2* value in the mid-ventricular septum was obtained by averaging T2* values in the mid anterior septum and the mid inferior septum. The T2* values measured depend on the MRI scanner and on the sequence used and different cut off values have been showed in literature, but it has never been reported a normal cut off value < 20 ms, that it has been conventionally assumed as ″conservative″ normal value [8,14,16,18]. Cardiac iron concentration (CIC) was derived from T2* values using the formula described by Carpenter et al. [10]. For the liver, the T2* value was calculated in a large region of interest (ROI) of standard dimension, chosen in a homogeneous area of parenchyma without blood vessels [15]. Care was taken to avoid the ROI placement in the posterior lateral (VII) and medial (VIII) segments, more prone to susceptibility artifacts [12]. For the liver an exponential + offset curve fitting model was applied [11]. A liver T2* < 9.2 ms was considered indicative of a significant load. Using the calibration curve introduced by Wood et al. [19], this cut-off corresponds to a LIC higher than 3 mg/g dry weight [20].

For the quantification of biventricular function parameters, steady-state free procession cine images were acquired during 8-second breath holds in sequential 8-mm short-axis slices (gap 0 mm) from the atrio-ventricular ring to the apex. Images were analysed in a standard way [13] using MASS® software (Medis, Leiden, The Netherlands). The inter-center variability for the quantification of cardiac function had been previously reported [21]. A LV EF ≥ 57% [22] and a right ventricular (RV) EF ≥ 55% [23] were considered normal.

### Statistical analysis

All data were analyzed using SPSS version 13.0 statistical package. Continuous variables were described as mean ± standard deviation (SD). Categorical variables were expressed as frequencies and percentages.

For the intra-treatment and the inter-treatment (between two groups) comparisons the changes between final and basal values were used for each quantitative variable.

The intra-treatment comparison was performed by univariate analysis of variance (ANOVA) with repeated measures.

The inter-treatment comparison for the baseline data and the changes between final and basal values was performed by the ANOVA. When the Levine test showed heteroscedasticy a log transformation or a Welch ANOVA was applied. If the log-transformation did not normalize the variable a non parametric Mann–Whitney test was used. The analysis of covariance (ANCOVA) was used to correct for variables (basal value, age, serum ferritin and MRI LIC) significantly different between the two treatment groups at the baseline and significantly associated to the dependent variable. χ2 test was performed for categorical baseline variables.

A P-value <0.05 was considered statistically significant.

## Results

### Characterization of the whole study population

The mean administered dosages of the chelators were: 1) DFP in combined regimen 61.9 ± 24.3 mg/kg body weight per day with a frequency of 6.1 ± 1.4 days/week and DFO in combined regimen 40.7 ± 6.0 mg/kg body weight per day with a frequency of 3.5 ± 1.1 days/week; 2) DFP in monotherapy 72.6 ± 12.5 mg/kg body weight with a frequency of 6.8 ± 0.9 days/week; 3) DFO in monotherapy 40.8 ± 6.5 mg/kg body weight via subcutaneous route on 5.5 ± 0.9 days/week. The percentage of patients with excellent/good levels of compliance to the active chelation treatment was not different between combined DFP+DFO group and DFP group (90.2% vs 94.9%; P=0.412) or between combined DFP+DFO group and DFO group (90.2% vs 95.9%; P=0.197). The mean time between the two CMR scans was comparable among groups (DFP+DFO 18.0 ± 1.5 months; DFP 17.7 ± 1.5 months; DFO 18.2 ± 1.3 months; P=0.264). The clinically and instrumentally relevant baseline findings in the three treatment groups are summarized in Table 1. The combined group was significantly younger and showed a significantly lower chelation starting age than the DFO group. The percentage of males was significantly lower in the combined group than in the DFP group. Mean serum ferritin levels were significantly higher in the combined group compared to both DFP and DFO groups. The global heart T2* as well as the mid-ventricular septum T2* values were significantly lower in the combined group compared to the DFP group and the DFO group. The number of segments with T2* value < 20 ms was significantly higher in the combined group than in DFP and DFO groups. The MRI LIC was significantly different in combined and DFO groups.

**Table 1:** Descriptive statistics of the treatment groups at baseline

	***P***	**Deferiprone group (n = 39)**	**Combined group (n = 51)**	**Deferoxamine group (n = 74)**	***P***
**Age (yrs)**	0.136	31.5 ± 5.3	29.6 ± 6.5	32.7 ± 8.5	0.032
**Male (%)**	0.022	69.2%	45.1%	48.6%	0.719
**Chel. starting age (yrs)**	0.499	5.1 ± 5.1	4.3 ± 4.1	6.6 ± 4.9	0.030
**Pre-transfusion Hb (g/dl)**	0.379	9.5 ± 0.7	9.6 ± 0.5	9.7 ± 0.6	0.213
**Ferritin (ng/l)**	0.001	941 ± 1541	1814 ± 1033	1093 ± 1256	0.001
**Global Heart (ms)**	0.0001	31.3 ± 11.3	21.5 ± 12.9	28.5 ± 10.7	0.002
**MRI CIC(mg/g dry w)**	0.001	0.94 ± 0.79	2.15 ± 2.38	1.03 ± 0.79	0.001
N seg. with T2* < 20 ms	0.001	3.9 ± 6.2	8.7± 7.0	4.4 ± 5.9	0.001
Mid septum T2* (ms)	0.0001	34.0 ± 13.0	22.3 ± 14.3	30.6 ± 12.8	0.001
**LV EF (%)**	0.643	61.8 ± 9.0	62.6 ± 7.0	61.6 ± 6.0	0.364
**LV EDVI (ml/m2)**	0.069	91.9 ± 20.0	84.7 ± 17.6	89.6 ± 20.2	0.164
**RV EF (%)**	0.336	59.9 ± 8.8	61.6 ± 6.8	60.9 ± 7.0	0.587
**RV EDVI (ml/m2)**	0.126	89.7 ± 19.9	83.8 ± 16.4	88.1 ± 20.5	0.210
Liver T2* (ms)	0.110	8.9 ± 8.0	6.3 ± 6.8	11.0 ± 7.5	0.0001
**MRI LIC(mg/g dry weight)**	0.330	8.2 ± 8.9	10.3 ± 10.1	5.3 ± 6.2	0.003

All values are quoted as mean ± SD. The P-values concern the comparison between combined and deferiprone groups and between combined and deferoxamine groups.

At baseline 108 patients had a global heart T2* value ≥ 20 ms and they were distributed in the treatment groups as follows: 25 in the combined group, 30 in the DFP group and 53 in the DFO group. At the follow-up the percentage of patients that maintained a normal global heart T2* value was comparable for combined and DFP groups (96.0% vs 100%, P=0.455) as well as for combined and DFO groups (96.0% vs 98.1%, P=0.541).

At baseline 133 patients had a normal LV EF. Of them, 43 were in the combined group (24 with a global heart T2* ≥ 20 ms), 31 in the DFP group (25 with a global heart T2* ≥ 20 ms) and 59 in the DFO group (44 with a global heart T2* ≥ 20 ms). The percentage of patients that maintained a normal LV EF was significantly lower in the combined group than in the DFP group (83.7% vs 100%, P=0.018) while it was not significantly different between combined and DFO groups (83.7% vs 83.1%, P=0.929). Among the patients with no significant myocardial iron burden at baseline (global heart T2* ≥ 20 ms) the percentage of patients that maintained a normal LV EF was significantly lower in the combined group than in the DFP group (79.2% vs 100%, P=0.022) while it was not significantly different between combined and DFO groups (79.2% vs 79.5%, P=1.0). Among the patients with a global heart T2* < 20 ms, the percentage of patients who maintained a normal LV EF was not significantly different between combined and DFP groups (89.5% vs 100%, P=1.0) or between combined and DFO groups (89.5% vs 93.3%, P=1.0).

At baseline 130 patients had a normal RV EF. Of them, 44 were in the combined group (21 with a global heart T2* ≥ 20 ms), 30 in the DFP group (26 with a global heart T2* ≥ 20 ms) and 56 in the DFO group (40 with a global heart T2* ≥ 20 ms). The percentage of patients who maintained a normal RV EF was not significantly different between combined and DFP groups (86.4% vs 96.7%, P=0.230) or between combined and DFO groups (86.4% vs 87.5%, P=0.867). Among the patients with a global heart T2* ≥ 20 ms, the percentage of patients who maintained a normal RV EF was not significantly different between combined and DFP groups (81.0% vs 96.2%, P=0.158) or between combined and DFO groups (81.0% vs 92.5%, P=0.220). Among the patients with a global heart T2* < 20 ms, the percentage of patients who maintained a normal RV EF was not significantly different between combined and DFP groups (91.3% vs 100%, P=1.0) or between combined and DFO groups (91.3% vs 75.0%, P=0.205).

### Baseline characteristics in patients with basal global heart T2* < 20 ms

At baseline 56 patients showed a global heart T2*< 20 ms: 26 in the combined group, 9 in the DFP group and 21 in the DFO group.

The mean administered dosages of the chelators were: 1) DFP in combined regimen 65.2 ± 22.5 mg/kg body weight per day with a frequency of 6.5 ± 1.1 days/week and DFO in combined regimen 41.7 ± 6.5 mg/kg body weight per day with a frequency of 3.3 ± 1.1 days/week; 2) DFP in monotherapy 79.2 ± 7.8 mg/kg body weight with a frequency of 6.6 ± 1.3 days/week; 3) DFO in monotherapy 42.2 ± 6.7 mg/kg body weight via subcutaneous route on 5.8 ± 0.7 days/week. The percentage of patients with excellent/good levels of compliance to the active chelation treatment was not different between combined and DFP groups (92.3% vs 88.9%, P=0.752) or between combined and DFO groups (92.3% vs 90.5%, P=0.823). The characteristics of these treatment subgroups at baseline are indicated in Table 2. The combined group was significantly younger than the DFO group. The serum ferritin levels were significantly higher in the combined group than in both DFP and DFO groups. The global heart as well as the mid-ventricular septum T2* values were significantly lower in the combined versus the DFO group, but not versus the DFP group. The combined group showed a LV EF comparable with both DFP and DFO groups and a RV EF comparable to the DFO group but significantly higher than the DFP group. The MRI LICs were significantly different in the combined than in both DFP and DFO groups.

**Table 2:** Baseline descriptive statistics of the treatment groups composed of patients with global heart T2* value < 20 ms

	***P***	**Deferiprone group (n = 9)**	**Combined group (n = 26)**	**Deferoxamine group (n = 21)**	***P***
**Age (yrs)**	0.358	29.7 ± 5.2	27.6 ± 5.7	32.8 ± 6.5	0.005
**Male (%)**	0.700	66.7%	53.8%	23.8%	0.072
**Chel. starting age (yrs)**	0.918	5.0 ± 3.5	5.3 ± 5.3	6.7 ± 6.0	0.504
**Mean pre-transfusion Hb (g/dl)**	0.180	9.9 ± 0.4	9.6 ± 0.5	9.8 ± 0.6	0.217
**Mean serum ferritin (ng/l)**	0.001	1475 ± 2753	2135 ± 966	1367 ± 988	<0.0001
**Global Heart (ms)**	0.112	12.8 ± 3.6	10.2 ± 4.3	14.4 ± 4.3	0.002
**MRI CIC** (**mg/g dry weight)**	0.096	2.20 ± 0.77	3.56 ± 2.65	1.99 ± 0.90	0.002
N seg. With T2* < 20 ms	0.502	14.8 ± 1.8	15.2 ± 1.7	13.0 ± 3.3	0.010
Mid septum T2* (ms)	0.194	13.3 ± 5.2	10.6 ± 5.4	14.6 ± 5.0	0.012
**LV EF (%)**	0.466	58.9 ± 5.9	61.1 ± 8.3	59.9 ± 6.8	0.583
**LV EDVI (ml/m2)**	0.749	90.2 ± 18.5	87.5 ± 22.2	89.9 ± 20.5	0.712
**RV EF (%)**	0.023	54.9 ± 5.7	60.2 ± 5.7	60.4 ± 6.0	0.927
**RV EDVI (ml/m2)**	0.737	89.3 ± 20.7	86.7 ± 19.7	86.1 ± 21.3	0.926
Liver T2* (ms)	0.011	7.5 ± 5.0	3.5 ± 3.5	8.2 ± 6.4	0.005
**MRI LIC (mg/g dry weight)**	0.018	8.4 ± 12.9	14.8 ± 11.8	6.8 ± 7.0	0.003

All values are quoted as mean ± SD. The P-values concern the comparison between combined and deferiprone groups and between combined and deferoxamine groups.

### Intra-treatment comparisons in patients with basal global heart T2* < 20 ms

The mean changes between final and basal values of each variable of interest are shown in Table 3.

**Table 3:** Inter-treatment (combined vs deferiprone and combined vs deferoxamine) prospective comparisons in patients with basal global heart T2* < 20 ms

	***P***	**Deferiprone group (n = 9)**	**Combined group (n = 26)**	**Deferoxamine group (n=21)**	***P***
**Mean Diff Serum Ferritin (ng/ml)**	0.005	−112 ± 241	−679 ± 835	−133 ± 575	0.017
Mean Diff Global Heart T2* (ms)	0.107	+8.8 ± 8.6	+4.5 ± 6.1	+3.7 ± 5.5	0.644
**Mean Diff MRI CIC** (**mg/g dry w)**	0.540	−0.36 ± 1.78	−0.75 ± 1.67	−0.35 ± 0.55	0.255
Mean Diff N seg. With T2* < 20 ms	0.100	−6.0 ± 5.6	−2.4 ± 3.8	−2.9 ± 3.7	0.638
Mean Diff Mid-Septum T2* (ms)	0.295	+6.1 ± 7.4	+3.3 ± 7.1	2.9 ± 5.6	0.841
**Mean Diff LV EF (%)**	0.181	+5.0 ± 6.4	+1.5 ± 6.7	+2.0 ± 6.5	0.802
**Mean DIFF LV EDVI (ml/m2)**	0.796	−6.0 ± 12.3	−4.6 ± 13.9	−7.7 ± 11.6	0.432
**Mean Diff RV EF (%)**	0.137	+6.8 ± 3.7	+3.2 ± 6.7	+0.2 ± 8.8	0.187
**Mean Diff RV EDVI (ml/m2)**	0.909	−6.9 ± 11.7	−7.5 ± 12.5	−5.8 ± 17.1	0.702
Mean Diff Liver T2* (ms)	0.010	+2.0 ± 7.5	+5.7 ± 6.9	+2.9 ± 4.2	0.026
**Mean Diff MRI LIC (mg/g/dw)**	0.009	−0.1 ± 3.2	−4.9 ± 6.1	−1.7 ± 2.8	0.024

The drop in mean serum ferritin levels was significant only in the combined group (P<0.0001).

**Figure 2:**
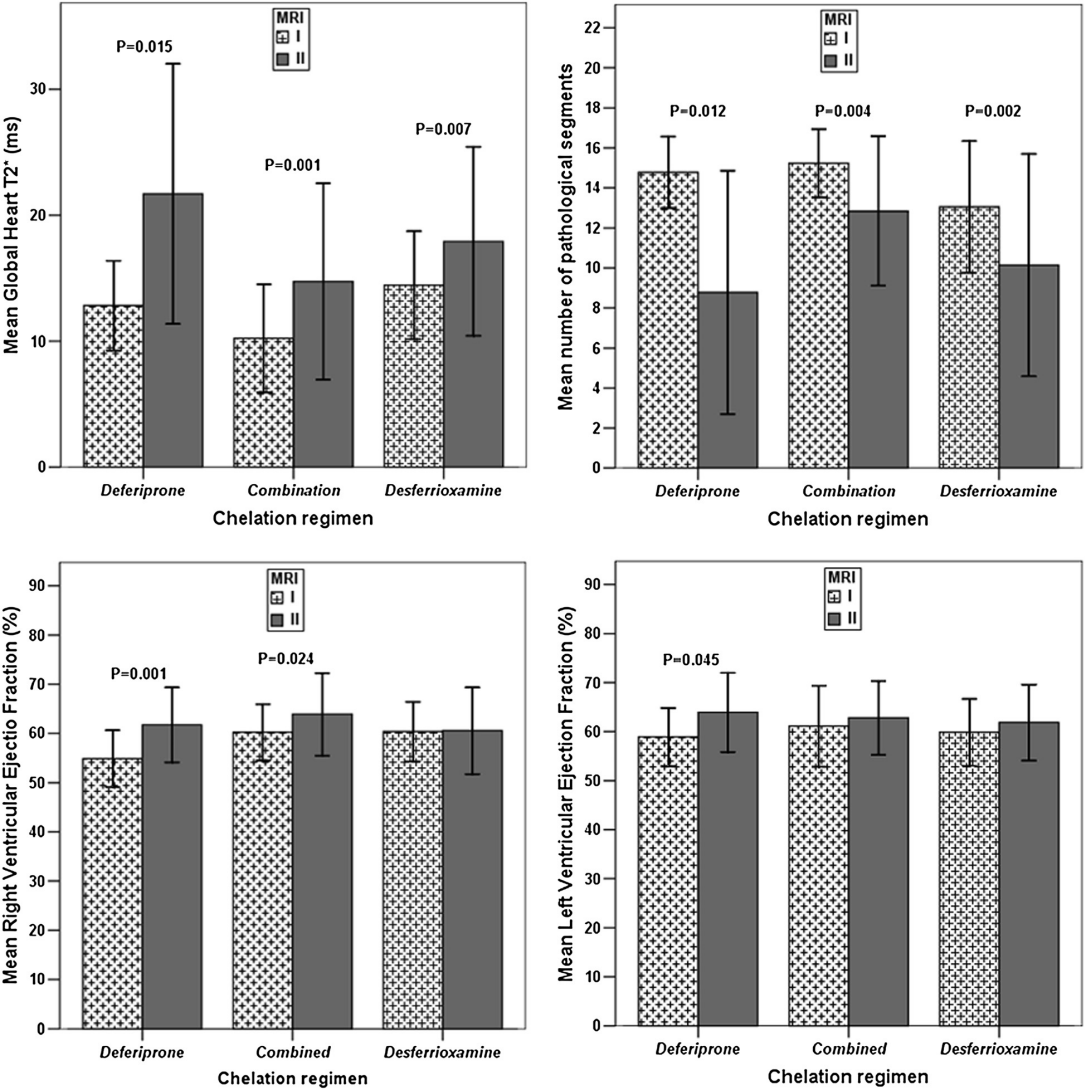
Intra-treatment comparison between final and basal values for heart iron and function in patients with basal global heart T2* value < 20 ms.

In all three groups there was a significant improvement in the global heart T2* values (combined: P=0.001; DFP: P=0.015 and DFO: P=0.007) and a significant reduction in the number of segments with an abnormal T2* value (combined: P=0.004; DFP: P=0.012 and DFO: P=0.002). Only in the DFP group there was a significant improvement in the LV EF (P=0.045) while the improvement in the RV EF was significant in both combined (P=0.024) and DFP (P=0.001) groups. No significant improvement in both left and right global systolic function was found in the DFO group. The reduction of the LV end-diastolic volume index (EDVI) was significant only in the DFO group (P=0.007) while the reduction of the RV EDVI was significant only in the combined group (P=0.006) (Figure 2).

The decrease of MRI LIC values was significant in the combined group (P=0.001) and in the DFO group (P=0.002).

### Inter-treatment comparisons in patients with basal global heart T2* < 20 ms

Table 3 shows the P-values referring to the inter-treatment comparisons.

The reduction in mean serum ferritin levels was significant greater in the combined group compared to both DFP and DFO groups, even adjusting for influential covariates (P=0.008 and P=0.003, respectively).

The changes in the global heart T2*, in the CIC, in the mid-ventricular septum T2* and in the number of segments with a T2* < 20 ms were not significantly different between the groups. After the correction for influential covariates the changes in global heart T2* values and CIC between combined and DFO groups became statistically different (P=0.014 and P=0.002, respectively).

No significant differences in both left and right global systolic function were found between the groups, also adjusting for influential covariates. Also repeating the ANCOVA for the LVEF including the basal global heart T2* values, the difference remained not significant (combined group vs DFP group: P=0.324; combined group vs DFO group: P=0.735). The changes in MRI LIC values were significantly higher in combined versus DFP and DFO groups. The significant difference between combined and DFO groups disappeared adjusting for influential covariates (P=0.102).

### Baseline characteristics in patients with basal MRI LIC > 3 mg/g/dw

At baseline 97 patients showed a MRI LIC > 3 mg/g/dw: 41 in the combined group, 23 in the DFP group and 33 in the DFO group.

The mean administered dosages of the chelators were: 1) DFP in combined regimen 62.9 ± 24.5 mg/kg body weight per day with a frequency of 6.1 ± 1.4 days/week and DFO in combined regimen 41.0 ± 6.2 mg/kg body weight per day with a frequency of 3.4 ± 1.1 days/week; 2) DFP in montherapy 75.8 ± 7.2 mg/kg body weight with a frequency of 6.6 ± 1.1 days/week; 3) DFO in monotherapy 41.7 ± 6.6 mg/kg body weight via subcutaneous route on 5.6 ± 1.1 days/week. The percentage of patients with excellent/good levels of compliance to the active chelation treatment was not different between combined and DFP groups (87.8% vs 91.3%, P=0.667) or between combined and DFO groups (87.8% vs 93.9%, P=0.370).

The characteristics of these treatment subgroups at baseline are reported in Table 4. The mean serum ferritin levels were significantly higher in the combined group than in both DFP and DFO groups.

**Table 4:** Baseline descriptive statistics of the treatment subgroups with liver T2* value < 9.2 ms

	***P***	**Deferiprone group (n = 23)**	**Combined group (n = 41)**	**Deferoxamine group (n =33)**	***P***
**Age (yrs)**	0.376	30.6 ± 6.1	28.9 ± 6.2	30.3 ± 8.3	0.439
**Male (%)**	0.108	69.6%	48.8%	51.5%	0.815
**Chel. starting age (yrs)**	0.548	5.1 ± 5.7	4.2 ± 4.3	5.4 ± 3.9	0.326
**Ferritin (ng/l)**	<0.0001	1347 ± 1782	2101 ± 944	1729 ± 1672	0.009
Liver T2* (ms)	0.733	3.3 ± 2.1	3.6 ± 2.3	4.0 ± 2.0	0.272
**MRI LIC (mg/g dry weight)**	0.940	12.5 ± 9.5	12.3 ± 10.2	9.6 ± 7.2	0.197

All values are quoted as mean ± SD.

### Intra- and inter-treatment comparisons in patients with basal MRI LIC > 3 mg/g/dw

**Figure 3:**
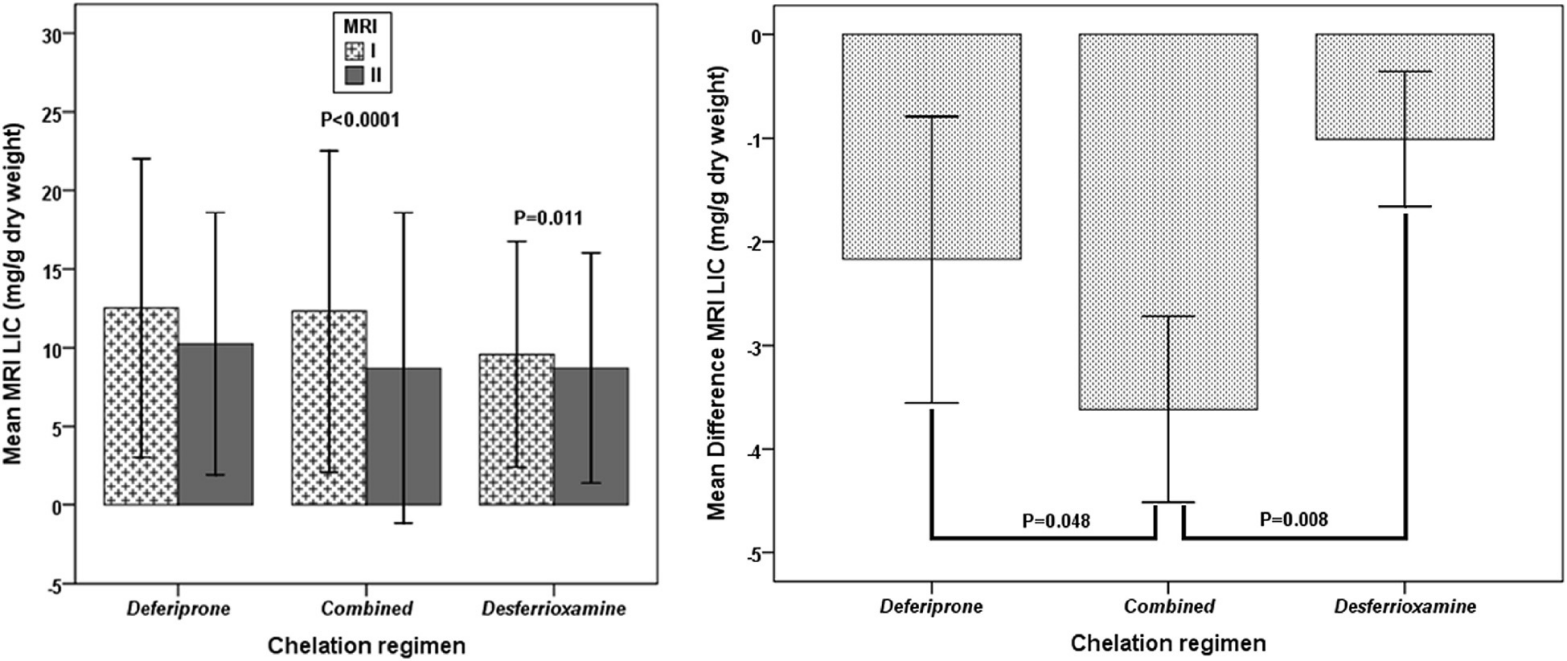
Inter-treatment and inter-treatment comparisons between final and basal values for MRI LIC values in patients with basal MRI LIC > 3 mg/g/dw.

The changes in mean serum ferritin values between the follow up and the baseline were −614 ± 851 ng/l in combined group, -8.7 ± 518 ng/l in DFP group and −214 ± 1074 ng/l in DFO group; with a statistical significance only for the combined group (P<0.0001). The MRI LIC decreased significantly in the combined group (−3.6 ± 5.8 mg/g/dw; P< 0.0001) and in the DFO group (−1.0 ± 3.7 mg/g/dw; P= 0.011). In the DFP group there was not a significant reduction in the MRI LIC (−2.3 ± 6.9 mg/g/dw; P= 0.071). In comparison to both DFP and DFO groups, the combined group showed a significant greater reduction in mean serum ferritin levels (P=0.019 and P=0.002, respectively), even adjusting for influential covariates (P=0.018 and P=0.007, respectively). The decrease in MRI LIC values was higher in the combined versus the DFP group, with a P-value near to the statistically significance (P=0.065); after adjustment for the basal mean serum ferritin levels the significance was reached (P=0.048). The decrease in MRI LIC values was significant higher in combined than in DFO group (P=0.008), even after adjustment for basal mean serum ferritin levels (P=0.008) (Figure 3).

## Discussion

Despite mounting scientific evidence coming from controlled trials on organ specific property of each chelator, currently in the clinical practice it is yet difficult to establish which is the most appropriate treatment for TM patients with and without significant iron overload. A mounting body of evidence highlights that combined DFO+DFP therapy is the most effective regimen against myocardial iron overload [4,5,7,24]. However, in TM patients no observational study prospectively evaluated in the real life the efficacy of the combined DFP+DFO therapy compared to both chelators in monotherapy. With the large numbers of patients included in its data base, the MIOT network is ideally situated to provide clear outcomes with respect to these questions. Moreover, in this project the myocardial iron burden is quantified by a segmental approach, that can be advantageous due to the heterogeneous myocardial iron distribution [25,26].

Out of the 164 patients considered in the current study, the 95% maintained between the two CMR scans the same chelation regimen that they had before the first CMR scan. The baseline characteristics of the whole study population (Table 1) reflect the current clinical practice. As assessed by mean serum ferritin levels and almost all CMR iron load parameters, heavily iron-loaded patients received more frequently combined DFP+DFO therapy. Such circumstance reflects the current evidence that combined DFP+DFO regimen seems to be the most effective therapy in removing iron, particularly appropriate in case of diffuse and/or severe iron overload. In this regards, in order to better evaluate prospectively the inter-treatment outcomes we introduced a covariance analysis and we adjusted for variables (basal value, age, serum ferritin and MRI LIC) significantly different between the groups at the baseline and significantly associated to the dependent variable. In fact, different baseline iron overload between groups could introduce bias by modulating the substrates for chelating agents.

All three groups showed high and comparable levels of compliance, confirming the intensive combined chelation regimen well-tolerated.

At 18 months of follow up in TM patients with no significant myocardial iron overload at baseline we provided evidence that all the regimens investigated could be effective as maintenance therapy regarding to the myocardial iron burden.

In all three groups with significant myocardial iron burden at baseline (global heart T2* < 20 ms) we found prospectively a significant improvement of the heart iron status both at global and segmental analysis (Figure 2).

Conversely, a significant improvement was found for both left and right global systolic function in the DFP group and only for the right global systolic function in the DFP+DFO group; no significant improvement in systolic heart function (left and/or right) was reported in patients treated only with DFO (Figure 2). Although the changes in cardiac function were not significant between groups, intra-treatment a significant improvement in the cardiac function (left and/or right ) was found only in the groups of patients where deferiprone was administered (in combined or in monotherapy). The benefit cannot be due to a total body iron reduction (serum ferritin and LIC), as DFP was not more effective than the others chelation regimens in this action, as also previously demonstrated [2,27]. The most likely explanation for the benefit on heart function is that the DFP improves myocardial mitochondrial function [28,29]. The DFP is an ideal chelating agent to cross cellular and subcellular membranes and it have an adequate affinity for binding labile iron as it is a small molecular weight bidentate with a neutral charge and an optimal partition coefficient to permeate membranes [30]. Our data about the significant higher efficacy in the DFP group (in particular in patients with an heart T2* ≥ 20 ms) in maintaining a normal heart function further supports the effect of the DFP on myocardial mitochondrial function independently to the iron accumulation/clearance. After the correction for influential covariates our data confirm in the clinical arena combined DFP+DFO regimen more effective than subcutaneous DFO in removing heart iron, as previously demonstrated by both one randomized clinical trial [4] and two observational studies [5,7].

Surprisingly, we did not find any additional efficacy in the combined DFP+DFO regimen versus the DFP in monotherapy in removing heart iron. No randomized or observational prospective data are available in the literature about the head to head comparison between combined DFP+DFO and DFP therapy. Anyway our observational prospective long term data seem to be consistent with one observational retrospective study that showed no additional effects of the combined DFP+DFO therapy versus the DFP monotherapy in removing heart iron [7]. In order to find an explanation for this datum, we compared the combined DFP+DFO therapy in the MIOT network and in the randomized controlled Tanner′s study [4] in the same baseline condition (patients with a baseline T2* value in the mid-ventricular septum ≥ 8 ms and ≤ 20 ms). The rate of clearance of myocardial iron was slightly lower in the combined therapy in the MIOT network than in that one of the Tanners′s study (difference between final and basal geometric means of the mid-ventricular septum T2* value: 4.46 ms versus 6.0 ms; ratio of geometric means: 1.35 vs 1.50, P=0.397). We found consistent lower DFO frequencies (days/week) in the MIOT combined regimen versus the Tanner′s trial (3.4 versus 5.0 days/week). One retrospective single center study showed that the modulation of the days per week in DFO combined therapy could influence the rate of removal of excess of iron load [31]. On the other hand, the variability in the number of DFO infusion days in combined regimen reflects how the combined DFP+DFO therapy has been used in clinical practice without a definite schedule and probably according to the severity of iron burden in the heart and in the liver. A similar observation was made in the previous cited observational analysis of the Greek group [7]. The lower efficacy of the combined DFP+DFO regimen in removing heart iron within the MIOT network could also justify the lack of a significant improvement in heart function in the combined group versus the DFO group found in our study population; conversely in the Tanner^′^s study was found a statistically significant greater improvement in the LVEF in the combined group compared to the DFO group [4].

No consistent differences were found between the MIOT data and the Tanner′s trial [4] in the global dosage, in the dosage per day or in the frequency per week for the DFP or in the global dosage or in the dosage per day for the DFO. In the same population in another prospective study published by Tanner et al. [5] about the combined therapy in patients with severe myocardial siderosis and impaired LV function, the dose of deferiprone prescribed was 73.9 ± 4.0 mg/kg/day for 7 days per week (patients^′^number 15) and at 12 months, this had been decreased to 65.7 ± 10.7 mg/kg/day, with dose reduction necessary in some case due to mild adverse events. The data were comparable with the administered dosages of the DFP in combined regimen in the MIOT Network (61.9 ± 24.3 mg/kg body weight per day with a frequency of 6.1 ± 1.4 days/week; patients^′^number 51). Nevertheless, it is recommended to prescribe and to maintain the maximum dosage of the DFP (75 mg/Kg/die) if tolerated, in order to avoid a significant impact on the efficacy of the combined therapy in the real life.

In TM patients with significant liver iron at baseline (MRI LICs higher than 3 mg/g dry) we found a significant reduction in LICs and serum ferritin levels in all treatments regimens. However, on this issue the inter-treatment analysis showed the combined regimen significantly more effective. These data on the liver accord with previous studies [4,6].

Additional prospective assessment of the ongoing MIOT data base will allow to further characterize the long-term comparative efficacy of these chelation regimens and to determine whether the observed difference will be maintained or will encounter an intrinsic limit in the heart chelating efficiency of each regimen. Moreover, the open enrollment in the MIOT network could increase the number of patients with significant cardiac iron burden at baseline, in particular in the DFP group. In fact, the small number of patients with significant myocardial iron overload at baseline in the DFP group represents one of the major limit of this study. At any rate luckily for our patients, it will be difficult to address this last issue due to the high and widespread quality of the TM health management in our country.

## Conclusions

In TM patients prospectively observed for 18 months, at the dosages used in the real world, combined DFP+DFO regimen and DFP in monotherapy were not significantly different in removing myocardial iron and improving heart function, while combined DFP+DFO therapy was more effective in the hepatic iron clearance. Combined DFP+DFO regimen confirmed its higher efficacy versus subcutaneous DFO in removing heart and liver iron, without an additional effect on the heart function. Further randomized controlled trials at fixed therapeutic schedules should be designed to verify these findings.

## Abbreviations

TM: Thalassemia major; CMR: Cardiovascular magnetic resonance; DFO: Deferoxamine; DFP: Deferiprone; DFX: Deferasirox; MRI: Magnetic resonance imaging; LV: Left ventricular; EF: Ejection fraction; LIC: Liver iron concentration; MIOT: Myocardial Iron Overload in Thalassemia; CIC: Cardiac iron concentration; RV: Right ventricular; SD: Standard deviation; EDVI: End-diastolic volume index.

## Competing interests

The MIOT project receives “no-profit support” from industrial sponsorships (Chiesi Farmaceutici S.p.A., ApoPharma Inc. and Bayer-Shering). This study was also supported by: “Ministero della Salute, fondi ex art. 12 D.Lgs. 502/92 e s.m.i., ricerca sanitaria finalizzata anno 2006” e “Fondazione L. Giambrone”. Dr. Pepe Alessia received speaker′s honoraria from Chiesi, ApoPharma Inc. and Novartis. The authors declare that they have no competing interests.

## Authors’ contributions

AP and PR conceived the study and wrote the paper. ML conceived the study. AM and GR performed the statistical analysis. LC, GDD, MS, PC, VC, MAR, AF, LP, MCP, AP, SC, MM, MM collected the data. LG was responsible for data collection. VP was responsible for data analysis. All authors contributed to critical revision and final approval of the version to be published.
